# SHP-1 Phosphatase Is a Critical Regulator in Preventing Natural Killer Cell Self-Killing

**DOI:** 10.1371/journal.pone.0044244

**Published:** 2012-08-31

**Authors:** Sajid Mahmood, Namita Kanwar, Jimmy Tran, Man-li Zhang, Sam K. P. Kung

**Affiliations:** 1 Department of Immunology, University of Manitoba, Winnipeg, Manitoba, Canada; 2 Manitoba Centre for Proteomics and Systems Biology, University of Manitoba, John Buhler Research Centre, Winnipeg, Manitoba, Canada; Centre de Recherche Public de la Santé (CRP-Santé), Luxembourg

## Abstract

Balance of signals generated from the engaged activating and inhibitory surface receptors regulates mature NK cell activities. The inhibitory receptors signal through immunoreceptor tyrosine based inhibitory motifs (ITIM), and recruit phosphatases such as SHP-1 to inhibit NK cell activation. To directly examine the importance of SHP-1 in regulating activities and cell fate of mature NK cells, we used our established lentiviral-based engineering protocol to knock down the SHP-1 protein expression in primary C57BL/6NCrl cells. Gene silencing of the SHP-1 in primary NK cells abrogated the ability of ITIM-containing NK inhibitory receptors to suppress the activation signals induced by NK1.1 activating receptors. We followed the fates of stably transduced SHP-1 silenced primary NK cells over a longer period of time in IL-2 containing cultures. We observed an impaired IL-2 induced proliferation in the SHP-1 knockdown NK cells. More interestingly, these “de-regulated” SHP-1 knockdown NK cells mediated specific self-killing in a real-time live cell microscopic imaging system we developed to study NK cell cytotoxicity in vitro. Selective target recognition of the SHP-1 knockdown NK cells revealed also possible involvement of the SHP-1 phosphatase in regulating other NK functions in mature NK cells.

## Introduction

Natural killer (NK) cells are lymphocytes that utilize a unique receptor recognition mechanism in mediating anti-viral and anti-tumor responses [1], [2]. These cells express a diverse repertoire of both activating and inhibitory surface receptors to facilitate their specific recognition of target cells [3], [4], [5]. Upon binding to their cognate ligands, activating receptors signal via the associated adaptor subunits that contain activation motifs, such as the immunoreceptor tyrosine-based activation (ITAM) and YXXM motifs, which facilitate the recruitment of downstream protein tyrosine kinases [6], [7]. Unlike activating receptors, inhibitory receptors express an Immunoreceptor Tyrosine-based Inhibitory Motif (ITIM) in their cytoplasmic portion [8], [9]. The ITIM (consensus sequence (I/V)xYxx(L/V) contains one tyrosine residue that is phosphorylated by activated Lck upon receptor engagement and ITIM clustering [10]. Recruitment and activation of the Src homology region 2 containing protein tyrosine phosphatase-1 (SHP-1) and/or SHP-2 via ITIM motifs provides a dominant inhibitory mechanism to prevent the induction of the stimulatory signalling cascade [9], [11], [12]. An integrated sum of signals generated from the combination of these engaged receptors determines the outcome of NK-target cell interactions, thereby ensuring NK specificity regulated by target cell expression of cognate NK receptors ligands [13], [14], [15], [16].

The importance of SHP-1 in transmitting inhibitory signals of the specific NK inhibitory receptors has been demonstrated [17]. Transient, over-expression of catalytically inactive dominant negative form of SHP-1 (dnSHP-1) in human and murine NK cells resulted in diminished KIR and Ly49-mediated inhibition in vitro [18], [19]. The latter is further supported by other studies of the mature NK cells of the transgenic animals or motheaten (*me*) and motheaten viable (*me^v^*) mice [19]. However, as SHP-1 signaling might be involved in both NK cell development and mature NK cell functions, analyses of mature NK cells of dnSHP-1 transgenic or SHP-1 deficient motheaten mice might represent NK defects associated with the loss of SHP-1 function in NK development and/or in mature NK cell functional regulation [18], [20], [21].

We established previously the utility of lentiviral vectors in mediating stable and efficient gene delivery in primary murine NK cells [16], [22]. In this study, we used our established in vitro lentiviral-based engineering protocol to down-regulate protein expression (and functions) of the SHP-1 at mature primary NK cell level to directly pinpoint the importance of SHP-1 molecule in regulating NK cell functions and viability at mature NK cell level.

## Results

### Efficient SHP-1 Gene Silencing in Primary Murine IL-2-activated NK Cells

**Figure 1:**
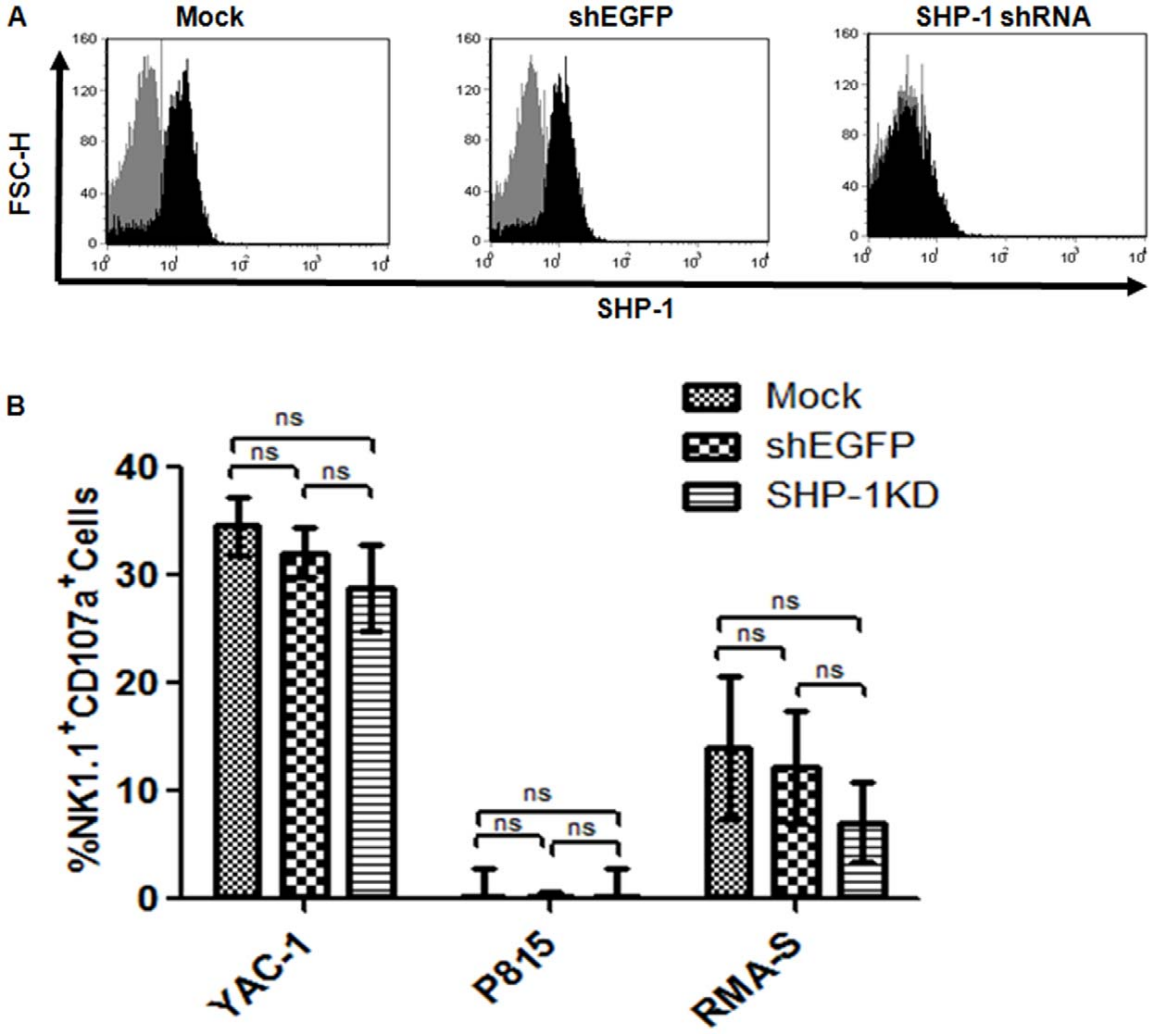
SHP-1 gene knockdown NK cells showed normal cytotoxicity towards prototypic tumor target cells. **A.** Efficient SHP-1 gene silencing in primary murine IL-2-activated NK cells. Purified C57BL/6NCrl IL-2 activated NK cells were transduced on two consecutive days with TRC lentiviral vectors and incubated for 3 days post-transduction. Transduced cells were puromycin selected for 48 hours followed by 3 days incubation. Cells were assayed for SHP-1 expression by intracellular staining with primary rabbit anti-SHP-1 and secondary anti-rabbit Alexa Fluor 488 antibodies in flow cytometry (Black). Secondary antibodies alone (Grey) was used as negative control. Data is representative of 3 experiments. B. IL-2 activated NK cells were transduced on two consecutive days with mock, shEGFP and SHP-1 shRNA and incubated for 3 more days. Transduced cells were puromycin-selected and used as effectors against Yac-1, P815 and RMA-S targets at E:T ratio 1∶1 in CD107a cytotoxicity assay. Background degranulation responses of the NK effectors (in the absence of target cells) were subtracted from the degranulation responses observed in the presence of the target cells. Data of three independent experiments were pooled together for statistical analyses. N.S., (p>0.05), non significant.

We searched the RNAi Consortium (TRC) lentiviral shRNA library (Open Biosystems, Thermo Fisher Scientific) for potential shRNA sequences targeting against the mouse SHP-1 gene (TRCN0000028964 - 68). TRC lentiviral vectors contain a puromycin selection marker that allowed us to study only the transduced cells of interest. Lentiviral vectors were produced from these clones and were screened in a murine EL4 T lymphoma cell line to identify the most potent shRNA sequence mediating stable and efficient SHP-1 gene knockdown. A TRC vector containing shRNA targeting the EGFP gene (designated as shEGFP) was used as a specificity control for SHP-1 gene knockdown. Analysis of the SHP-1 protein expressions by Western Blot and intracellular staining in flow cytometry revealed that the TRCN0000028966 clone mediated the most efficient SHP-1 silencing in EL-4 cells (Figure S1). We therefore used the TRCN0000028966 clone (in short, SHP-1-shRNA) in the silencing of the SHP-1 gene in primary NK cells. As shown in Figure 1A, the SHP-1-shRNA clone mediated about 85% SHP-1 down-regulation as compared to the mock transduced control and the shEGFP-transduced specificity control. We therefore confirmed successful gene silencing of the SHP-1 protein expression in primary mouse LAK cells.

### Gene Silencing of SHP-1 Expression did not Affect NK Cells-mediated Cytotoxicity Towards Prototypic Tumor Target Cells

Recognition of target cells and the induction of cell cytotoxicity were regulated by the integration of the activities of various kinases or phosphatases recruited by the activating and inhibitory receptors in NK cells [23], [24]. As the SHP-1 phosphatase was associated with a majority of mouse inhibitory NK receptors [24], we examined whether the SHP-1 knockdown NK cells were hyper-responsive in their cytotoxic responses against prototypic tumor targets (YAC-1, P815 and RMA-S). To test this, Mock transduced and the shEGFP-transduced LAK cells were used as controls, and the SHP-1-shRNA-transduced LAK cells were used as effectors in a lysosomal-associated membrane protein-1 (LAMP-1 or CD107a) flow cytometric assay for cell-mediated cytotoxicity [25], [26], [27], [28], [29]. CD107a has been recently identified as a marker of degranulation (associated with perforin and granzyme release) on human CD8+ cytotoxic T and NK lymphocytes upon antigen stimulation and target interactions. This non-radioactive assay has been validated against standard ^51^Cr release cytotoxicity assay, and widely used in the analyses of cell-mediated cytotoxicity [25], [26], [27], [28], [29]. We did not observe any statistical difference in the YAC-1 killing whether the mock transduced, the shEGFP-transduced or the SHP-1-shRNA transduced NK cells were used as effector cells in the assay (Figure 1B). Similarly, no statistical differences in their cytotoxicity were observed when other tumor targets, such as P815 (a relatively NK-resistant tumor cell) or RMA-S (a NK-sensitive MHC class I deficient cell) were used in the assay (Figure 1B).

### SHP-1 Knockdown NK Cells Showed Loss of Inhibition in the Antibody Induced Redirected Lysis Assay

**Figure 2:**
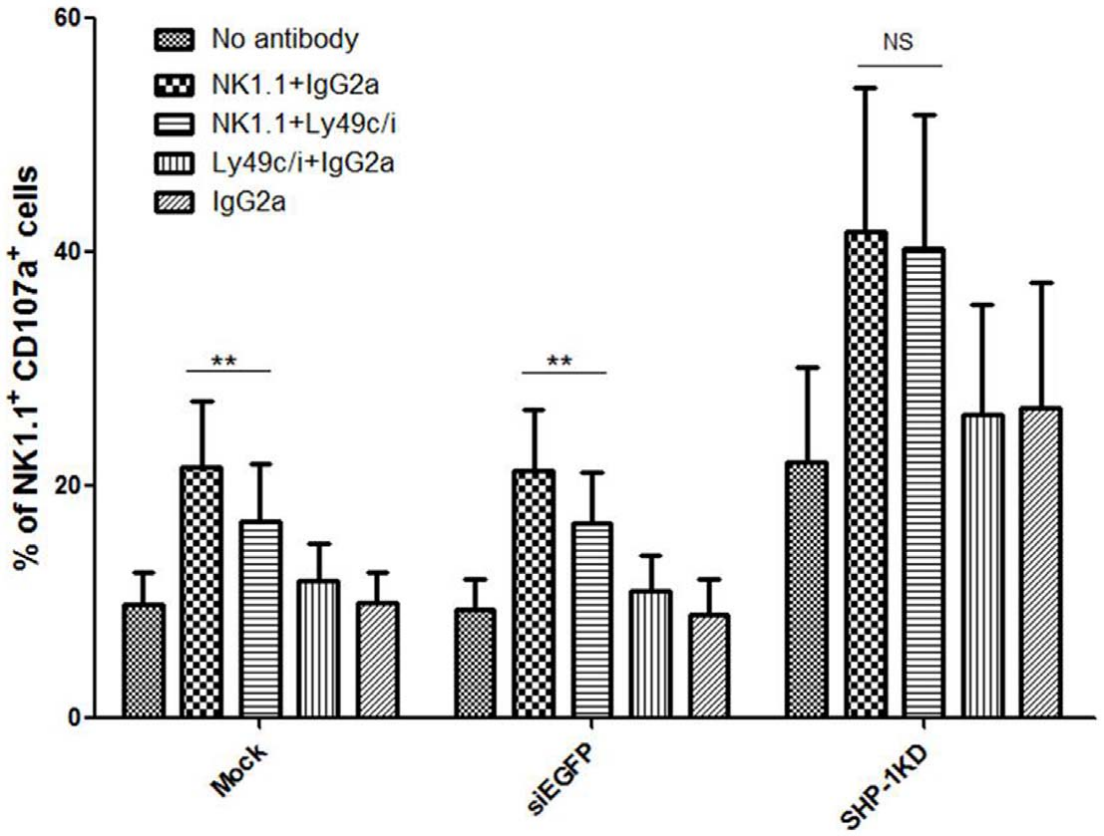
SHP-1 knockdown NK cells showed loss of inhibition in the antibody induced redirected lysis assay. Mock, shEGFP and SHP-1 shRNA transduced and puromycin selected LAK cells incubated with purified antibodies for 20-minutes. Effector:target (P815) cells (1∶1 ratio) in V-shaped 96-well plate incubated for 5 & 4 hours in the presence of CD107a antibody and monensin respectively. Percentages of NK1.1^+^CD107^+^ cells were calculated and statistically analyzed using graph pad prism software. A p-value of <0.05 was considered statistically significant. Data is representative of 5 experiments.

In an antibody-induced redirected lysis (AIRL) assay, specific NK receptor function/signalling is analyzed using an antibody specific to the NK receptor, and a target cell (eg. P815, Daudi) that bears a Fc receptor (FcR) molecule on the cell surface. The fragment antigen-binding (Fab) portion of an antibody specific to a NK receptor of interest (activation or inhibitory) binds specifically to the receptor on the surface of the NK cells while its Fc portion binds to the target cell Fc receptor that provides a crosslinking effect [30], [31], [32]. Depending on the activiation or inhibitory nature of the NK receptor, such crosslinking triggered the receptor signaling/function to induce or suppress, respectively, the cytotoxic activity of the NK cells against the FcR-bearing P815 target cells in the assay. Addition of an antibody specific to the activating NK1.1 receptor stimulated C57BL/6NCrl NK cells to kill the previously relative resistant P815 target cells [30]. Co-engagement of an inhibitory receptor (such as Ly49C/I), however, overrode the stimulatory signals induced by the NK1.1 receptor in a SHP-1 dependent manner [33], [34], [35], [36], [37]. We therefore used this assay system to determine the impact of the SHP-1 gene silencing on the ability of the specific inhibitory NK receptor to exhibit a dominant inhibitory signal over the activation signal triggered by the NK1.1 engagement. Similar to the mock transduced NK cells, the shEGFP-transduced and the SHP-1-shRNA-transduced NK cells were induced to lyse the P815 target cells in the presence of anti-NK1.1 mAb. Co-engagement of the Ly49C/I inhibitory receptors on the mock and shEGFP-transduced cells inhibited the activation induced by the anti-NK1.1 mAb. In contrast, we observed that the SHP-1 knockdown NK cells lost their ability to inhibit the NK1.1-induced activation upon Ly49C/I receptor engagement (Figure 2). Collectively, the data demonstrated that lentiviral transductions and expression of an irrelevant shRNA did not impair NK function in the redirected lysis assay. Specific SHP-1 gene silencing in primary NK cells exerted a functional impact on NK inhibitory receptor signalling/function.

### Stable SHP-1 Gene Silencing in Primary NK Cells Resulted in Impaired IL-2 Induced NK Cell Proliferation and Loss of Cell Viability

**Figure 3:**
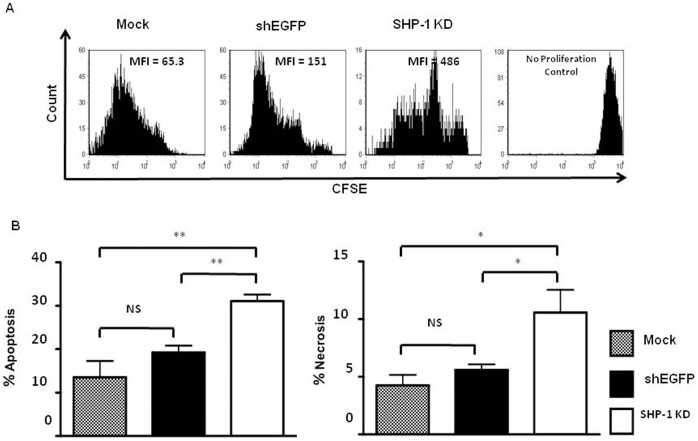
Stable SHP-1 gene knockdown in primary NK cells resulted in impaired IL-2 induced NK cell proliferation. A. Mock, shEGFP and SHP-1 shRNA transduced and puromycin selected LAK cells were labeled with carboxyfluorescein succinimidyl ester (CFSE) and cultured them again in IL-2 supplemented media. On day 7 post CFSE staining, cells were surface stained with NK1.1 antibody. Samples were acquired and gated on NK1.1^+^ population for CFSE dilution analysis. B. Loss of cell viability in the SHP-1 knockdown NK cells. Mock, shEGFP and SHP-1 shRNA transduced and puromycin selected LAK cells were for apoptosis and necrosis by stained with Annexin V and 7-amino actinomycin D (7AAD) respectively in flow cytometery. Data is representative of 3 experiments. NS, non-significant; *, *p*<0.05; **, *p*<0.01.

Lentiviral vectors transduce and integrate into host genomes of NK cells without observable impaired NK functions and loss of cell viability, thus supporting persistent expression of introduced genetic materials in the modified NK cells [38]. Our ability to generate stable SHP-1 gene silencing in primary NK cells allowed us to examine further the long-term impact of the impaired SHP-1 function in vitro. We observed that the SHP-1 knockdown NK cells consistently yielded a lower total cell number at the end of the IL-2 culture, when compared to the shEGFP-transduced control cells. We therefore examined directly the proliferation potential and cell viability in the mock, shEGFP-transduced and SHP-1-shRNA-transduced NK cells using carboxyfluorescein succinimidyl ester (CFSE) dilution and AnnexinV/7AAD staining respectively [39], [40]. In the CFSE dilution assay, each round of cellular proliferation reduces the mean fluorescence intensity (MFI) in half because the CFSE is partitioned equally to each daughter cell [41]. We observed that the SHP-1 knockdown NK cells had a higher MFI of 486 compared to 65 and 151 for mock and EGFP-transduced NK cells respectively (Figure 3A), suggesting fewer rounds of cell proliferation in SHP-1 transduced NK cells. To assay for cell viability, we used mock transduced LAK cells as a control for normal apoptosis and necrosis, and shEGFP-transduced LAK cells as a specificity control for the SHP-1-transduced LAK cells. We observed that 31.1% of the SHP-1-shRNA-transduced NK cells were undergoing apoptosis, as compared to 13.6% of the mock and 19.3% of the EGFP-transduced NK cells (Figure 3B). 10.6% of the SHP-1-shRNA-transduced NK cells were undergoing necrosis compared to 4.3% of the mock and 5.6% of the shEGFP-transduced NK cells. The SHP-1 knockdown NK cells showed a significant increase in both cellular apoptosis (*p*<0.01) and necrosis (*p*<0.05) when compared to the mock and the shEGFP-transduced NK cells.

### SHP-1 Knockdown NK Cells Showed Increased “Spontaneous” Degranulation

**Figure 4:**
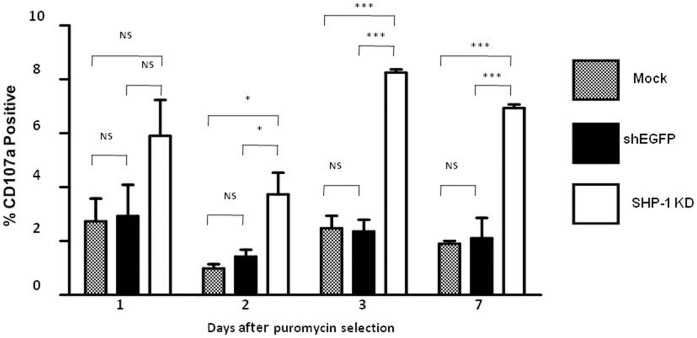
SHP-1 gene knockdown NK cells showed increased spontaneous degranulation. Mock, shEGFP and SHP-1 shRNA transduced and puromycin selected LAK cells were incubated in IL-2 supplemented media for 1,2,3 or 7 days. Cells were then used in a CD107a degranulation assay. Briefly, cells were incubated with CD107a antibody and monensin in 5 ml centrifuge tubes for 5 hours at 37°C before analysis in flow cytometry. Data is representative of 3 experiments. NS, non-significant; *, *p*<0.05; ***, *p*<0.0001.

The balance of signals generated from simultaneous ligand interaction with inhibitory and activating receptors regulates NK-mediated self-non-self discrimination [42]. Normal cells predominantly express ligands for inhibitory receptors. This skews the balance of receptor signalling towards attenuation of NK cell activity leading to self-tolerance [43]. We examined whether gene silencing of SHP-1 promoted their “spontaneous” cytotoxic activity of the SHP-1 knockdown NK cells. We measured CD107a degranulation in the SHP-1 knockdown NK cells and the Mock and shEGFP-transduced control NK cells, in the absence of any target cells, over a period of 7 days in vitro. On day 1, 5.9% of the SHP-1 knockdown NK cells showed degranulation as compared to 2.7% and 2.9% degranulation from the mock and the shEGFP-transduced NK cells, respectively (Figure 4). The differences however, were not statistically significant. On day 2, 3.7% of the SHP-1 knockdown NK cells showed the degranulation activity as compared to 1.0% and 1.4% degranulation from the mock and the shEGFP-transduced NK cells, respectively and the differences were significant (*p*<0.05). On day 3 and day 7, the SHP-1-shRNA-transduced NK cells continued to show a significant increase in CD107a degranulation as compared to the mock and the shEGFP-transduced NK cells (*p*<0.0001). We observed that the SHP-1 knockdown NK cells underwent increased “spontaneous” degranulation in vitro.

### Real-time Imaging of Spontaneous Killing of the SHP-1-knockdown NK Cells in vitro

The observed increase in spontaneous degranulation suggested active NK cell-mediated killing in the SHP-1 knockdown NK cells. To directly visualize these events in vitro, we used 7AAD to detect dying cells as they would take up 7AAD and appeared red under microscope. We first used the untransduced primary NK cells of C57BL/6NCrl and the prototypic YAC-1 targets to validate the system. Cell tracker green CMFDA dye was used to label target cells (YAC-1) in the experiments that required discrimination of target cells from effector cells. The CMFDA-labelled YAC-1 cells were mixed with unlabelled primary NK cells at 1∶1 ratio in the presence of 7AAD. Specific interactions and killing of YAC-1 cells events were observed by tracking a green target cell turned red (upon incorporation of 7AAD) over time. We observed that the unlabelled primary NK cells actively engaged with the labelled YAC-1 target cells (Figure S2, Video S1). YAC-1 cells were subsequently eliminated and were visualized in the form of reduction in green fluorescence signal from the cytoplasmic dye, and an increase in red fluorescence due to the uptake of nuclear stain 7AAD (Video S1). We also imaged unlabelled NK cells alone in the presence of 7AAD to confirm no non-specific killing of the primary NK cells by each other over time (Video S2). We also confirmed that the CMFDA dye did not affect the viability of the labelled target cells within the time line used in the imaging procedure because no 7AAD uptake was observed in the labelled YAC-1 cells over time (Video S3).

**Figure 5:**
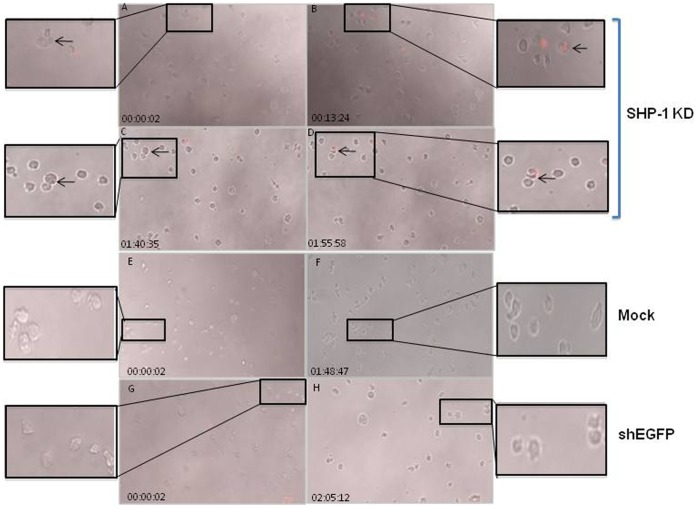
Real-time imaging of spontaneous killing of the SHP-1-knockdown NK cells in vitro. Mock, shEGFP transduced and SHP-1 shRNA transduced and puromycin selected purified NK cells resuspended in Hanks Buffered Salt Solution (HBSS) with 10% FCS and 50 U/ml IL-2 containing 7AAD. Cells were imaged every 25 seconds for up to 5 hours using 10X magnification objective on a Zeiss Observer 710 station. The images in the figures were excised from live cell imaging movies (Videos S4, S5, S6) at different time points. Dying cells appeared red due to 7AAD staining. Frames A–B, C–D: events at different time points of the real-time live cell imaging. Frames B, D represented late time points of the same cells after 10–20 minutes of interactions (as noted in A and C respectively, indicated by the arrows). The inset images showed the zoom-in regions of interest for clarity.

Next, we used this cell imaging platform to track cytotoxic activities of the mock transduced, shEGFP-transduced and SHP-1-shRNA transduced NK cells in vitro. Unlabelled primary NK cells, mock transduced cells and the SHP-1 knockdown cells were imaged in the presence of 7AAD for up to 5 hours using 10X magnification objective on a Zeiss Observer 710 station at the rate of 4 images per minute. We did not observe conjugate formation or active killing in the mock and shEGFP-transduced controls (Figure 5)(Videos S4, S5). In contrast, stable cell contacts and conjugate formation were detected in the SHP-1 knockdown NK cells. Real-time tracking of these same cells at a later time point demonstrated that the SHP-1 knockdown NK cells recognized each other as potential targets, which subsequently led to killing and 7AAD uptake over time (Figure 5)(Video S6).

**Figure 6:**
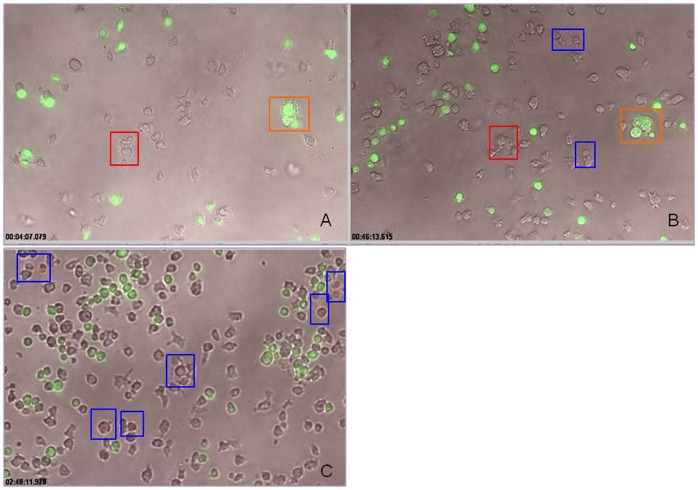
Specific recognition of the SHP-1 gene knockdown NK cells as targets. SHP-1 shRNA transduced and puromycin selected purified NK cells mixed with green CMFDA-labelled normal C57BL/6NCrl NK cells at 1∶1 ratio, resuspended in Hanks Buffered Salt Solution (HBSS) with 10% FCS and 50 U/ml IL-2 containing 7AAD. Cells were imaged every 25 seconds for up to 5 hours using 10X magnification objective on a Zeiss Observer 710 station. The images in the figure were taken from the live cell imaging videos (Video S7) at different time points. Red box in insert A indicated real cell conjugate formation between two unlabelled NK cells. These conjugated cells can be tracked 46 minutes later (red box, insert B) to show subsequent cytolysis as the dying cells took up the 7-AAD dye. Orange box indicated transient aggregates that disintegrated at later time points, and showed no evidence of cytolysis. At late time-points (such as 150 minutes of imaging), more apoptotic cells (red) were observed in the unlabelled SHP-1 knockdown NK cells (Blue box).

We performed a mixing experiment in which unlabeled SHP-1 knockdown NK cells were co-cultured with green CMFDA-labeled normal primary NK cells at 1∶1 ratio. As described above, we observed active engagement of the SHP-1 knockdown cells followed by cytolytic activity (Figures 5, 6). However, we did not observe any stable cell-cell conjugate formation between the SHP-1 knockdown and normal NK cells (Figure 6). Also, we did not observe labeled green normal NK cells turned red in this mixing experiment (Video S7), demonstrating specific recognition and subsequent killing of the SHP-1 knockdown NK cells as non-self targets by the other SHP-1 knockdown NK cells.

## Discussion

In the current study, we examined directly the importance of the SHP-1 phosphatase in regulating mature primary NK cell functions. We were successful in identifying potent shRNA target sequence against mouse SHP-1 gene (Figure 1). The SHP-1 gene silencing in primary NK cells abrogated the ability of the ITIM-containing NK inhibitory receptors to suppress activation signals induced by the NK1.1 activating receptor (Figure 2). For the first time we were able to follow the fates of the stably transduced SHP-1 gene silenced, primary NK cells over a longer period of time in IL-2 containing cultures. We observed an impaired IL-2 induced proliferation (Figure 3A), higher level of cell death (Figure 3B) and an increased in spontaneous CD107a degranulation in the SHP-1 knockdown NK cultures over time (Figure 4). Using a real-time live cell microscopic imaging system, we visualized that these “de-regulated” NK cells were mediating specific self-killing in vitro (Figure 5, 6).

The ability to introduce stable gene silencing of the SHP-1 molecules in mature splenic NK cells provided us a tool to pinpoint the functional importance of the SHP-1 molecules at mature NK cell levels. It is of importance that we observed that the SHP-1 silenced NK cells lost the ability to inhibit the NK1.1-induced activation upon Ly49C/I inhibitory receptor engagement (Figure 2) – first, it established that our RNA interference approach could prove useful in studying protein function(s) in primary NK cells; second, the importance of the SHP-1 phosphatase in specific inhibitory receptor signalling could not be compensated by other phosphatases or signalling molecules; third, despite the presence of some “residual” amount of the SHP-1 protein in the SHP-1-shRNA transduced cells, these SHP-1 silenced NK cells had a functional phenotype similar to the NK cells of the SHP-1 dominant negative transgenic or SHP-1 deficient motheaten mice [19], [20], [21]. Our current data therefore demonstrated a definitive role of SHP-1 in regulating mature NK cell function(s) that is independent of potential effect of SHP-1 signaling on NK cell development/education.

The apparent lack of undesirable toxicity associated with lentiviral vector transduction facilitated long-term analyses of the stably modified NK cells in vitro. Strikingly, we observed an increase in spontaneous CD107a degranulation and direct NK cell self-killing in the SHP-1 silenced NK cultures over time (Figure 4, 5). To directly visualize the cell killing events in vitro, we developed a real-time live cell microscopic imaging system to assay for NK cell cytotoxicity. This imaging system allowed real-time visual monitoring of cytolytic events that endpoint assays (such as Chromium release cytotoxicity) did not support. When used with a CMFDA cell tracker dye, it captured two confirmatory indicators of cell killing - the loss of cytoplasmic cell tracker dye and subsequent uptake of the 7AAD viability dye in the dying cells. In addition, one can further correlate cytolytic potentials with the other parameters (such as their migration ability, morphology) that have a direct impact on the cytolytic ability.

It is conceivable that the self-killing of the SHP-1-gene silenced NK cells in vitro may account for increased cell death observed in vitro, and indirectly the impaired cell proliferation that is due to a significant killing of a number of activated, proliferating NK cells in culture. However, it might also be possible that these observations were independent events that revealed other previously unappreciated functional roles of the SHP-1 phosphatase in NK cell regulation. Of note, our data seems to suggest also that the gene silencing of the SHP-1 phosphatase induced NK receptor ligands and/or adhesion molecules that promote specific NK cell target recognition. First, the SHP-1-gene silenced NK cells were not rendered hyper-reactive to all targets, due to a “global” impairment of NK inhibitory receptors signalling and therefore a tipping of the NK receptors signalling balance towards activation. We observed comparable cytotoxic activities in prototypic NK cell tumor targets (YAC-1, P815 and RMA-S)(Figure 1). Second, in a mixing experiment in which normal NK cells were added in the SHP-1-knockdown NK cell culture as potential targets, we observed that the SHP-1 knockdown NK cells selectively “recognized” the SHP-1 knockdown NK cells in the real-time live cell microscopy (Figure 6). Third, phenotypic analysis of various receptor molecules including NK1.1, KLRG1, CD11b, Ly49C/I/F/H, DX5, CD27, CD69, NKG2D & NKp46 showed that the SHP-1 knockdown NK cells were selective in upregulating some of the known activating receptors. We observed only enhanced expression levels of activation marker (CD69) and NK activating receptor (NKG2D and NKp46) in the SHP-1 knockdown NK cells when compared to the mock, the shEGFP-transduced controls (Figure S3). It remained to be determined whether a specific subset of NK cells was responsible for the recognition and subsequent killing of the SHP-1 knockdown NK cells; also which receptor/ligand is involved in such recognition. Analyses of MHC class I (Db and Kb) and Rae-1 expression on the SHP-1 knockdown NK cells and the control NK cells did not reveal any differences in their surface expressions (Figures S4, S5).

Our previous work established the efficiency and feasibility of using VSV-G pseudotyped lentiviral vectors in genetic engineering of primary NK cells [38]. In a gain-of-function study, we used the NKR-P1B^SJL^ inhibitory receptor as a model receptor to formally demonstrate that over-expression of an NK receptor on primary NK cells was able to manipulate balance of NK receptor signalling, and thus NK-target cell specificity [16]. Here, using SHP-1 phosphatase as a gene silencing target in a loss-of-function study, we demonstrated that lentiviral vectors were efficient in delivering specific shRNA (RNA interference) in primary NK cells. NK cells that were transduced by the shEGFP irrelevant silencing control vectors did not show any observable differences in functional activities when compared to the mock (untransduced) NK cells. It therefore established that our transduction and puromycin selection procedures, de novo production of shRNA inside NK cells, and the lentiviral vector integrations has little/no observable impact on the functional activities of the transduced NK cells.

In summary, our current work established a stable gene-silencing platform that can be applied to study any protein of interest in primary NK cells (mouse or human) [44], [45]. The development of the real-time imaging system to study NK cell cytotoxicity will prove useful in visualizing steps (such as on/off target cell binding and conjugate formation) involved in target cells lysis. Future examinations of the SHP-1 knockdown NK cells will reveal further molecular pathways regulated by the SHP-1 phosphatase in NK cells.

## Materials and Methods

### Cell Lines

293T cells used for virus production and titration were cultured in Iscove’s modified Dulbecco’s medium (IMDM) (HyClone, Logan, Utah) supplemented with 10% fetal bovine serum (FBS) (HyClone) and 1% penicillin/streptomycin/L-glutamate (PSG) (Gibco, Grand Island, NY). YAC-1, RMA-S and P815 cell lines were cultured in Roswell Park Memorial Institute (RPMI)-1640 (HyClone) supplemented with 10% FBS, 1% PSG, and 1.6 mM 2-ME (Sigma, St. Louis, MO).

### Lentiviral Vector Transduction of Primary NK Cells

C57BL/6NCrl mice were purchased from Animal Care Services (The University of Manitoba). The University of Manitoba’s Review Board has approved all animal studies. Primary NK cells were isolated from spleen using the EasySep Mouse NK Negative Selection Kit (StemCell Technologies, Vancouver, BC). ShRNA clones against mouse SHP-1 (TRCN0000028964-68) were obtained from the RNAi consortium (TRC) Lentiviral shRNA library. Production of the VSV-G pseudotyped lentiviral vectors and the transduction of primary NK cells were described previously [22], [46]. In brief, resting NK cells (1.0×10^6^/group) were mock transduced or transduced with the shEGFP control vector and clone TRCN0000028966 (28966 in short or SHP-1 knockdown (SHP-1 KD) at a multiplicity of infection (MOI) of 20 in 48-well culture plates and centrifuged at 2000 RPM for 2 hours at room temperature. A second round of transduction was conducted after 24 hours to further enhance the transduction efficiency. Transduced NK cells were cultured in 0.5 ml of supplemented RPMI media containing 1000 units/ml of IL-2 for 3 days before puromycin (4 µg/ml) selection for 48 hours. Cells were washed and cultured further in mouse medium containing IL-2 for 2 more days before final analysis.

### Flow Cytometry

Purified and conjugated antibodies against NK1.1, Ly49 C/I, IgG2a, (San Diego, CA). KLRG1, CD11b, Ly49C/I/F/H, DX5, CD27, CD69, Rae-1, K^b^, D^b^, NKG2D, NKp46 and CD107a were purchased from BD Bioscience (San Diego, CA). Annexin V-PE (BioVision, Mountain View, CA) and 7-amino actinomycin D (7-AAD) (Sigma, St. Louis, MO) were used in apoptosis assay. Rabbit anti-SHP-1 antibody (Upstate) and anti-rabbit Alexa Fluor 488 (Biolegend, San Diego, CA) were used in intracellular staining of SHP-1. Samples acquisition was performed on a FACS Canto (BD) using Diva software (BD) and data was analyzed using Flowjo software.

### ^51^Chromium Release Cytotoxicity Assay

NK cytotoxicity was assessed in a standard 4-hour ^51^Cr-release assay against YAC-1, P815, and RMA-S tumor cells. The supernatant was counted in liquid scintillation and luminescence counter (Trilux 1450 Microbeta). Percentage of target cells killed was calculated using the formula, %Killing = [(experimental release – spontaneous release)/(total release – spontaneous release)] × 100.

### CD107a Degranulation Assay

Effector and target cells (0.2×10^6^ each, 1∶1 ratio) were mixed in 5 ml centrifuge tubes to a final volume of 0.2 ml. 1 µl of CD107a-PE (BD, San Diego, CA) antibody was added, mixed and incubated at 37°C and 5% CO_2_ for one hour. 6 µg of monensin (Sigma) (2 mg/ml in methanol) was added, tubes were mixed and incubated at 37°C and 5% CO_2_ for an additional 4 hours. Cells were washed and then surfaced stained with NK1.1 APC as described previously [16] before acquisition in flow cytometry. In the antibody induced redirected lysis (AIRL) assay, mock, shEGFP- and SHP-1 shRNA-transduced IL-2 activated NK cells were incubated with purified NK1.1, Ly49 C/I, IgG2a antibodies (5 µg/ml) for 20 minutes at room temperature, as follows: Panel A: No antibody; Panel B: NK1.1+IgG2a; Panel C: NK1.1+Ly49 C/I; Panel D: Ly49 C/I + IgG2a; Panel E: IgG2a. The cells were washed with mouse medium to get rid of unbound antibodies. Effector and P815 target cells (0.1×10^6^ each, 1∶1 ratio) were mixed in V-shaped 96-well plate to a final volume of 0.2 ml. 1 µl of CD107a PE antibody was added, mixed and incubated at 37°C and 5% CO_2_ for 1 h. 6 µg of monensin was added, mixed and incubated at 37°^C^ and 5% CO_2_ for 4 hours. Cells were harvested, washed and surface stained for the expression of NK1.1-APC before acquisition in flow cytometry. Percentage of NK1.1^+^CD107a^+^ cells was calculated for final statistical analysis.

### Carboxyfluorescein Succinimidyl Ester (CFSE) Labelling

NK cells (0.75×10^6^) were washed and resuspended in 5 ml of 1× PBS. 5 ml of 3 µM CFSE stain (Molecular Probe) were added to the cells and incubated at room temperature for 7 minutes. 5 ml of 10% FBS was added and mixed very gently. Cells were pelleted at 1500 RPM for 10 minutes at room temperature. Cell pellet was washed again with supplemented RPMI 1640 medium before resuspending the cells in 1 ml of IL-2 (1000 units/ml) supplemented RPMI 1640 medium. The cells were transferred into a 24-well plate and cultured at 37°C and 5% CO_2_.

### Live Cell Imaging of NK Cytotoxicity

Labelling media was prepared fresh by adding Cell Tracker ^(^™^)^ Green CMFDA dye (Invitrogen Catalog No. C2925) in 5 ml serum free RPMI-1640 media containing 1.0% BSA to a final concentration of 1.0 µm. Target cells were collected by centrifugation at 1000 rpm for 5 minutes, resuspended in 5 ml labelling media and incubated for 15 minutes at 37°C and 5% CO_2_. Cell suspension was centrifuged at 1000 rpm for 5 minutes to remove excess dye containing media and washed once with fresh RPMI-1640 media containing 10% FCS. Washed target cells were resuspended in 5 ml pre-warmed RPMI-1640 media containing 10% FCS and incubated for another 30 minutes at 37°C and 5% CO_2_. The labelled target cells were spun down at 1000 RPM for 5 minutes, washed once with media containing 10% FCS and pellets were finally resuspended in Hanks Buffered Salt Solution (HBSS) with 10% FCS and 50 U/ml IL-2. Effector cells and labelled target cells were mixed at Effector: Target ratio of 1∶1 in a final volume of 500 µl. 5 µl 7AAD was added to the cell suspension and was placed in one well of a 24-well plate. The cells were allowed to settle down on the bottom of the wells for 2 minutes and image acquisition was immediately started every 25 seconds for up to 5 hours using 10X magnification objective on a Zeiss Observer 710 station while maintaining the cells at 37°C. The images in the figures were excised from live cell imaging movies at different time points. Movies and images were analysed using AxioVision software version 4.8.1. Dying cells were characterized by the loss of cytoplasmic dye and subsequent uptake of 7AAD nuclear stain that visually appeared as reduction in green and increase in red fluorescence of the dying target cells.

### Statistical Analysis

Data were analyzed statistically using the Graph Pad Prism. Two-way ANOVA (Bonferroni post-test) and two-tailed student t-test were used to analyze the statistically significance of the results. A p-value of <0.05 was considered statistically significant.

## Supporting Information

Figure S1 **Efficient SHP-1 gene knocked down in EL-4 cells.** EL-4 cells were transduced on two consecutive days by the “spin protocol,” with TRC lentiviral vectors and incubated for 3 days post-transduction. Transduced cells were puromycin selected for 48 hours followed by 3 days incubation. Cells were assayed for SHP-1 expression by western blot and intracellular staining with primary rabbit anti-SHP-1 and secondary anti-rabbit Alexa Fluor 488 antibodies in flow cytometry. Data is representative of 2 experiments.
(TIF)

Figure S2 **Validation of the real-time in vitro imaging of NK target interactions, conjugate formation and apoptosis of target cells.** A live cell in-vitro imaging system developed where events were imaged every 25 seconds using 10X magnification objective on a Zeiss Observer 710 station. Images in the figure were taken from supplementary videos (Videos S1, S2, S3). Unlabeled primary NK cells alone (pNK) were cultured in Hanks Buffered Salt Solution (HBSS) with 10% FCS and 50 U/ml IL-2 in the presence of 7AAD. Early and late time points images, showing no evidence of non-specific killing in the culture (A)(Video S2). Similarly, cell tracker green CMFDA dye was used to label target cells (YAC-1) and analyzed in live cell in-vitro imaging system over time in the presence of 7AAD (B)(Video S3). Viability of YAC-1 appeared uncompromised and all cells eventually maintained green florescence throughout acquisition time frames. (C) Real conjugate of NK cells and its prototypic YAC-1 target cells were formed, and subsequent led to apoptosis in YAC-1 cells. The green CMFDA-labelled YAC-1 cells were mixed with unlabelled primary NK cells at 1∶1 ratio in HBSS with 10% FCS and 50 U/ml IL-2 containing 7AAD. Images showed stable conjugate formations between NK and YAC-1 cells. Target cells lost the intensity of green fluorescence and picked 7AAD staining, an indication of apoptosis, as shown by arrows at late time point events (Video S1).
(TIF)

Figure S3 **Analysis of the cell surface receptor expression in the SHP-1 knockdown NK cells.** Mock, shEGFP transduced and SHP-1 shRNA transduced and puromycin selected NK cells were subjected to phenotypic analysis by standard flow cytometry using antibodies against NK1.1, KLRG1, CD11b, Ly49C/I/F/H, DX5, CD27, CD69, NKG2D and NKp46 surface receptors. SHP-1-shRNA transduced NK cells showed enhanced expression level of CD69-activation marker and NK activating receptor molecules like NKG2D and NKp46 as compared to the mock and the shEGFP-transduced controls.
(TIF)

Figure S4 **SHP-1 knockdown NK cells exhibited comparable normal MHC-1 expression.** Mock, shEGFP-transduced and SHP-1 shRNA- transduced NK cells were tested for MHC-1 surface expression. Cells were surface stained with PE-conjugated anti-H-2K^b^ and anti-H-2K^b^ monoclonal antibodies, and analyzed in flow cytometry.
(TIFF)

Figure S5 **SHP-1 knockdown NK cells exhibited comparable Rae-1 expression.** Mock, shEGFP-transduced and SHP-1 shRNA-transduced NK cells were surface stained for the expression of Rae-1 in flow cytometry. SHP-1 knockdown NK-cells, when compared to the mock and shEGFP- transduced controls, demonstrated no observable difference in their Rae-1 expression. YAC-1 cells were used as positive control of Rae-1 staining.
(TIFF)

Video S1 **Real-time imaging of YAC-1 killing by activated NK cells – YAC-1 killing.** The green CMFDA-labelled YAC-1 cells were mixed with unlabelled primary NK cells at 1∶1 ratio in HBSS with 10% FCS and 50 U/ml IL-2 containing 7AAD. Images were acquired every 25 seconds using 10X magnification objective on a Zeiss Observer 710 station. Active conjugations between NK and YAC-1 cells were observed. YAC-1 target cells become apoptotic, red in color to 7AAD uptake and lost green fluorescence in the culture over time.
(MOV)

Video S2 **Real-time imaging of YAC-1 killing by activated NK cells – NK cells only control.** Unlabaled primary NK were cultured in HBSS with 10% FCS and 50 U/ml IL-2 in the presence of 7AAD. Images were acquired every 25 seconds using 10X magnification objective on a Zeiss Observer 710 station. No evidence of non-specific NK killing observed during image acquisition.
(MOV)

Video S3 **Real-time imaging of YAC-1 killing by activated NK cells – YAC-1 only control.** Green CMFDA labelled YAC-1 target cells were set for live cell imaging analysis under the same experimental and imaging conditions as described previously. All YAC-1 target cells, in the absence of NK cells, showed a complete viability (no 7AAD staining) without any loss of green fluorescence during acquisition.
(MOV)

Video S4 **Real-time imaging of spontaneous killing of the SHP-1-knockdown NK cells in vitro – Mock transduced control cells.** Mock transduced primary NK cells were cultured in HBSS with 10% FCS and 50 U/ml IL-2 in the presence of 7AAD, and were imaged for up to 5 hours using 10X magnification objective on a Zeiss Observer 710 station. No cell-cell conjugate formation and subsequent killing activities were observed.
(MOV)

Video S5 **Real-time imaging of spontaneous killing of the SHP-1-knockdown NK cells in vitro – shEGFP-transduced control cells.** ShEGFP-transduced primary NK-cells were puromycin-selected, cultured in HBSS with 10% FCS and 50 U/ml IL-2 in the presence of 7AAD, and imaged for up to 5 hours using 10X magnification objective on a Zeiss Observer 710 station. No cell-cell conjugate formation and subsequent killing activities were observed.
(MOV)

Video S6 **Real-time imaging of spontaneous killing of the SHP-1-knockdown NK cells in vitro - SHP-1 shRNA transduced NK cells.** SHP-1 shRNA-transduced primary NK-cells were puromycin-selected, cultured in HBSS with 10% FCS and 50 U/ml IL-2 in the presence of 7AAD, and imaged for up to 5 hours using 10X magnification objective on a Zeiss Observer 710 station. The SHP-1 knockdown NK cells showed higher tendency for stable conjugate formation with other cells. Conjugated cells subsequently mediated self-killing and turned red due to 7AAD staining.
(MOV)

Video S7 **Real-time imaging of the SHP-1 knockdown NK cells recognizing each other as cellular targets.** We mixed unlabelled SHP-1 shRNA transduced primary NK cells with the CMFDA-labeled normal NK-cells (green) in a coculture, and imaged cytotoxicity for up to 5 hours in the presence of 7AAD, as described previously. Unlabelled SHP-1 silenced NK cells interacted with both kinds of cells in the culture over time. However, stable conjugate formations and subsequent cytolysis were only observed when the SHP-1 silenced NK cells interacted with the SHP-1 silenced NK cells. The dying unlabelled NK cells turned red due to the uptake of 7AAD. We observed also interactions (also aggregates) of normal NK cells; however, we did not observe that the normal NK cells (green) lost their green florescence intensity and evidence of cytolysis (uptake of 7AAD) in the 5-hours live cell imaging analysis.
(MOV)
