# Supplementary figures and images for: SHP-1 Phosphatase Is a Critical Regulator in Preventing Natural Killer Cell Self-Killing
Source: PLoS One. 2012 Aug 31;7(8):e44244. doi: 10.1371/journal.pone.0044244 (PMC3432062; doi:10.1371/journal.pone.0044244)

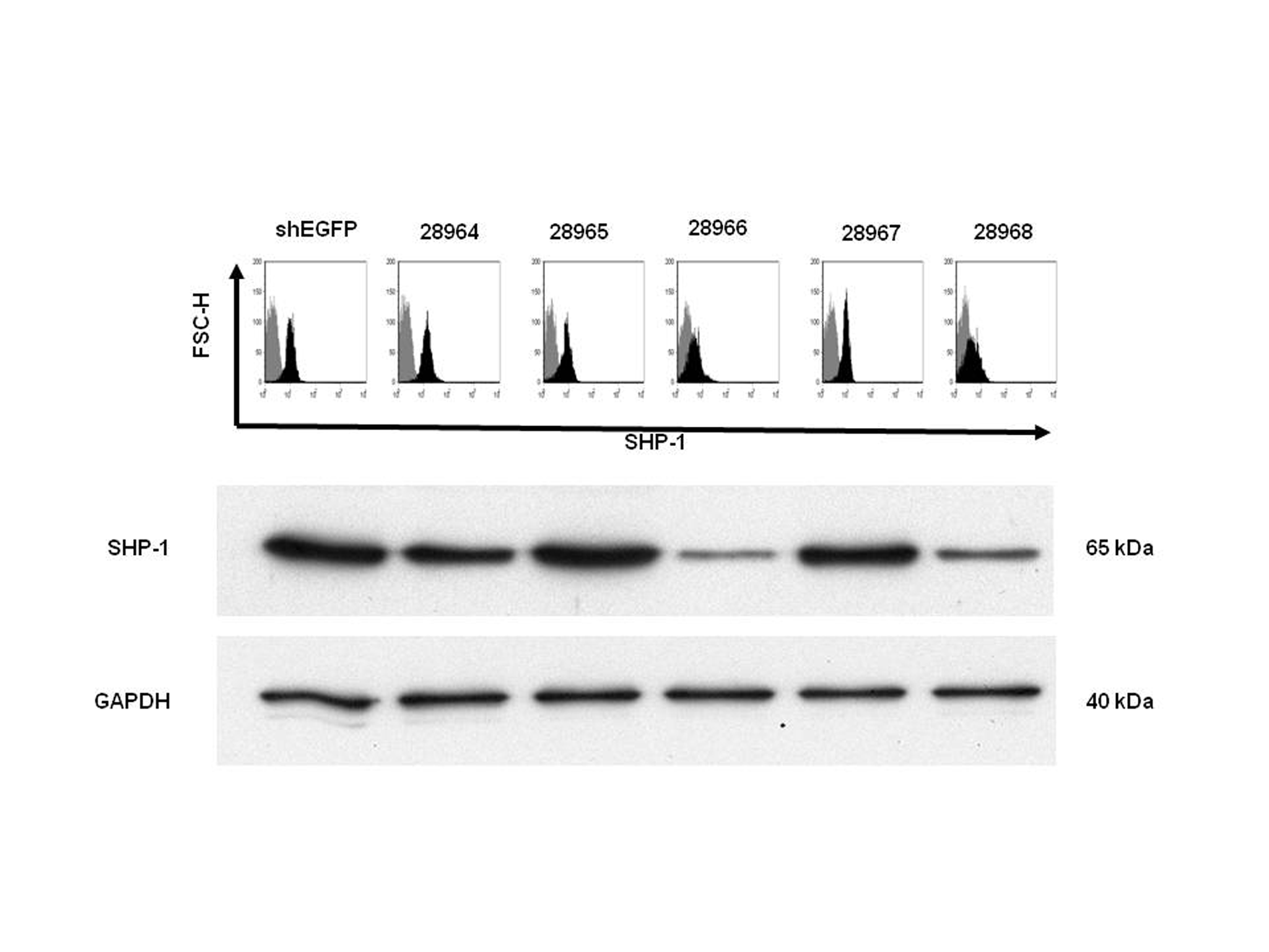

Supplement: Figure S1 — Efficient SHP-1 gene knocked down in EL-4 cells. EL-4 cells were transduced on two consecutive days by the “spin protocol,” with TRC lentiviral vectors and incubated for 3 days post-transduction. Transduced cells were puromycin selected for 48 hours followed by 3 days incubation. Cells were assayed for SHP-1 expression by western blot and intracellular staining with primary rabbit anti-SHP-1 and secondary anti-rabbit Alexa Fluor 488 antibodies in flow cytometry. Data is representative of 2 experiments. (TIF) [file pone.0044244.s001.tif]

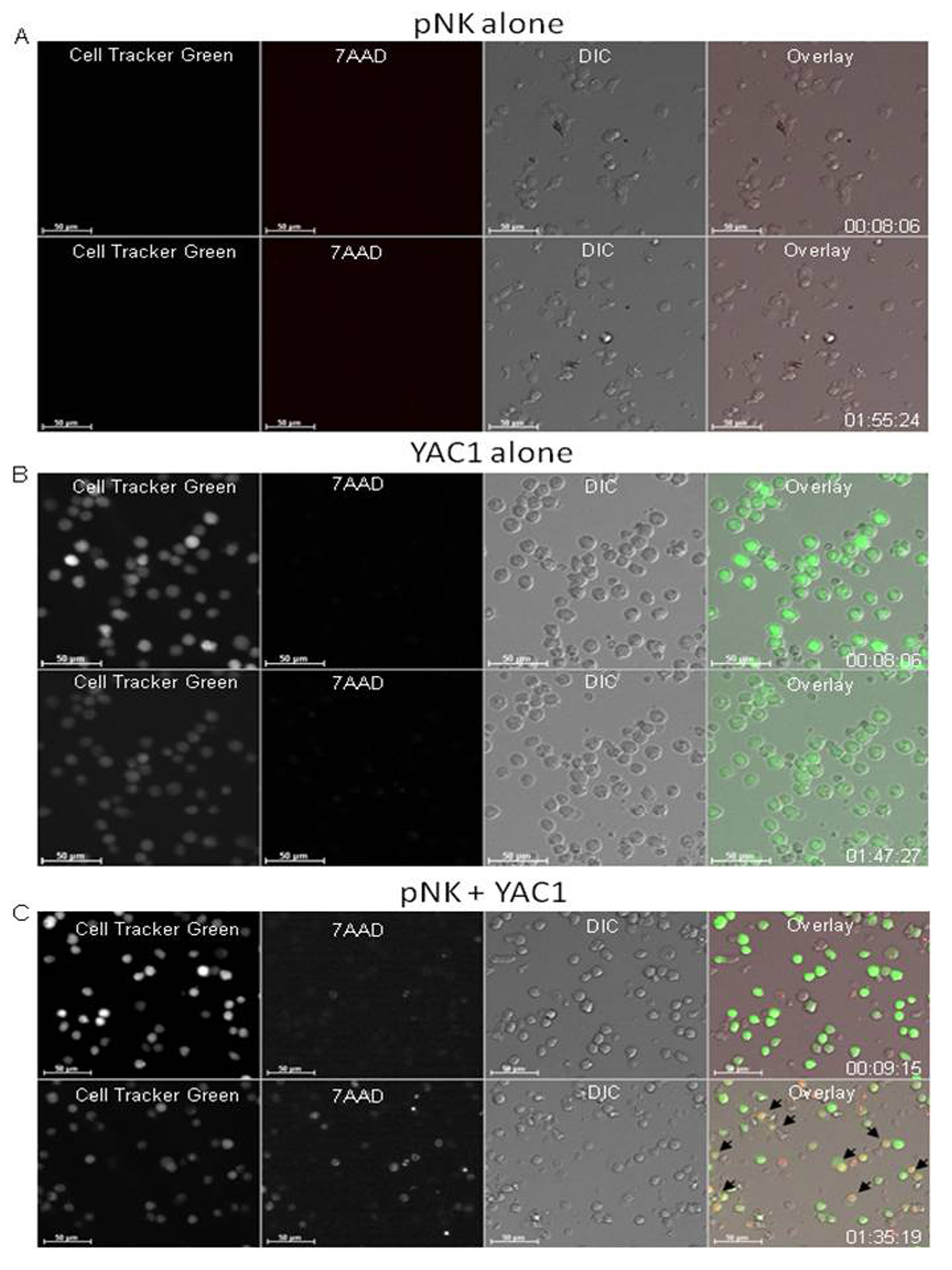

Supplement: Figure S2 — Validation of the real-time in vitro imaging of NK target interactions, conjugate formation and apoptosis of target cells. A live cell in-vitro imaging system developed where events were imaged every 25 seconds using 10X magnification objective on a Zeiss Observer 710 station. Images in the figure were taken from supplementary videos (Videos S1, S2, S3). Unlabeled primary NK cells alone (pNK) were cultured in Hanks Buffered Salt Solution (HBSS) with 10% FCS and 50 U/ml IL-2 in the presence of 7AAD. Early and late time points images, showing no evidence of non-specific killing in the culture (A)(Video S2). Similarly, cell tracker green CMFDA dye was used to label target cells (YAC-1) and analyzed in live cell in-vitro imaging system over time in the presence of 7AAD (B)(Video S3). Viability of YAC-1 appeared uncompromised and all cells eventually maintained green florescence throughout acquisition time frames. (C) Real conjugate of NK cells and its prototypic YAC-1 target cells were formed, and subsequent led to apoptosis in YAC-1 cells. The green CMFDA-labelled YAC-1 cells were mixed with unlabelled primary NK cells at 1∶1 ratio in HBSS with 10% FCS and 50 U/ml IL-2 containing 7AAD. Images showed stable conjugate formations between NK and YAC-1 cells. Target cells lost the intensity of green fluorescence and picked 7AAD staining, an indication of apoptosis, as shown by arrows at late time point events (Video S1). (TIF) [file pone.0044244.s002.tif]

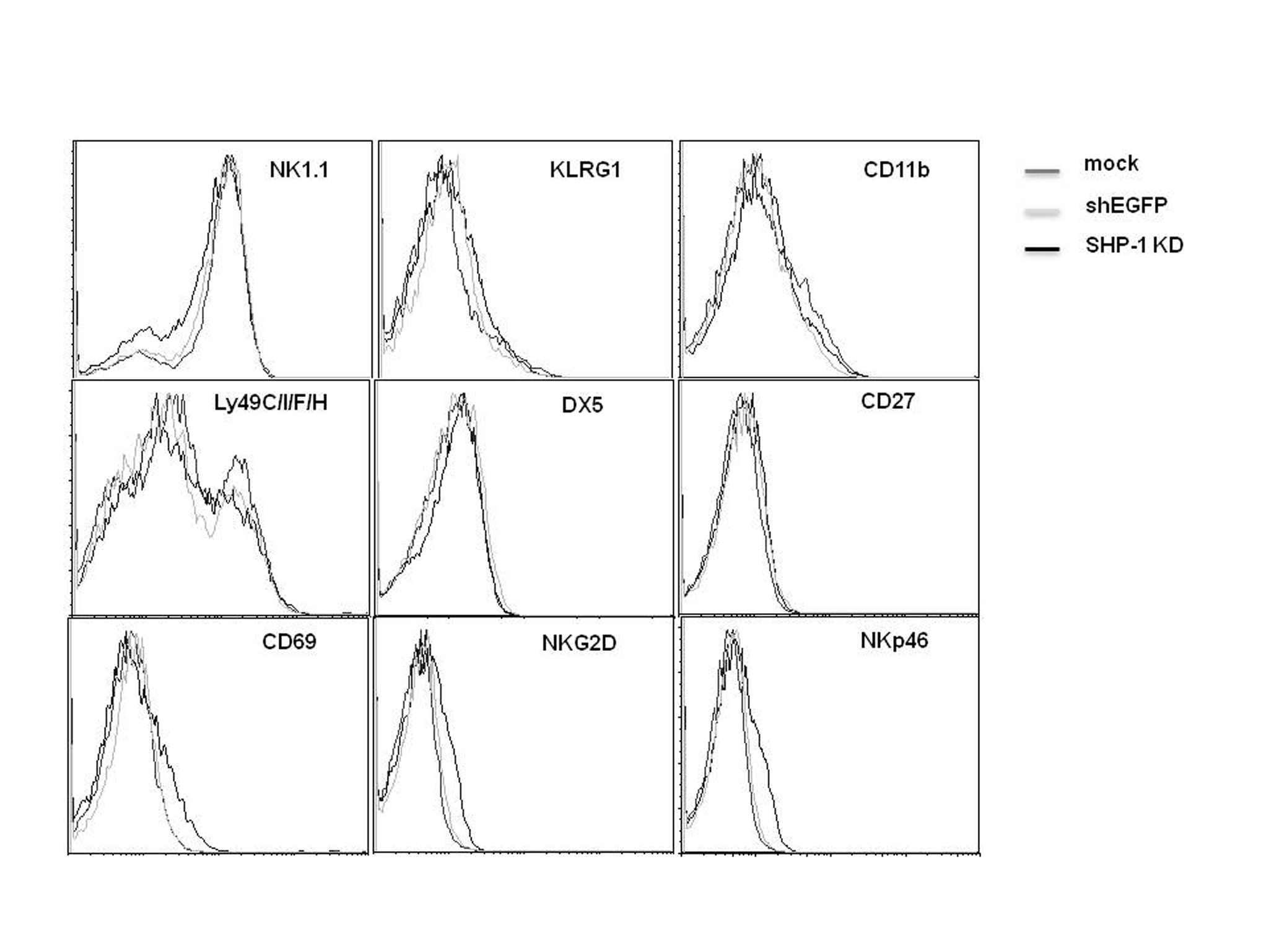

Supplement: Figure S3 — Analysis of the cell surface receptor expression in the SHP-1 knockdown NK cells. Mock, shEGFP transduced and SHP-1 shRNA transduced and puromycin selected NK cells were subjected to phenotypic analysis by standard flow cytometry using antibodies against NK1.1, KLRG1, CD11b, Ly49C/I/F/H, DX5, CD27, CD69, NKG2D and NKp46 surface receptors. SHP-1-shRNA transduced NK cells showed enhanced expression level of CD69-activation marker and NK activating receptor molecules like NKG2D and NKp46 as compared to the mock and the shEGFP-transduced controls. (TIF) [file pone.0044244.s003.tif]

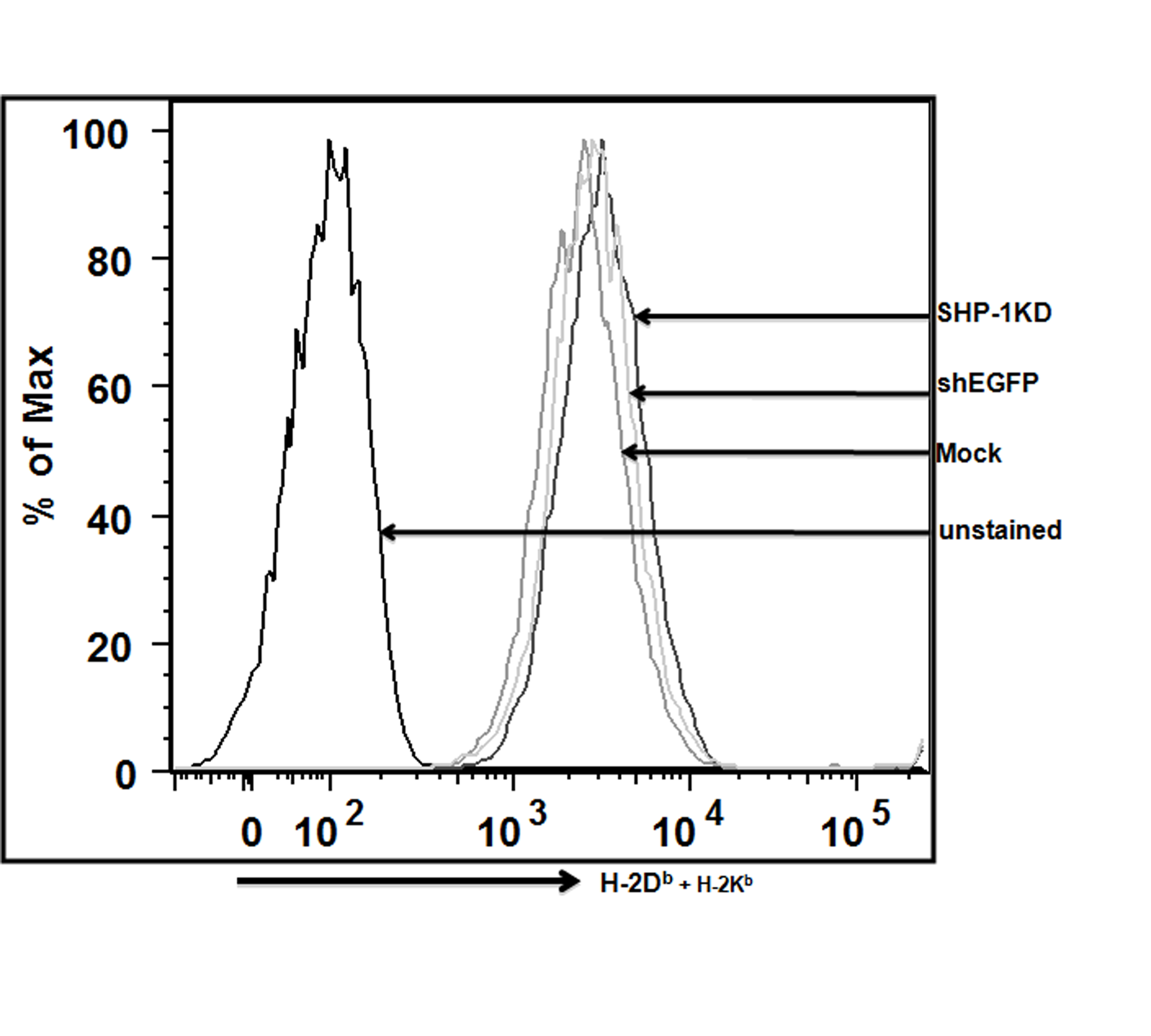

Supplement: Figure S4 — SHP-1 knockdown NK cells exhibited comparable normal MHC-1 expression. Mock, shEGFP-transduced and SHP-1 shRNA- transduced NK cells were tested for MHC-1 surface expression. Cells were surface stained with PE-conjugated anti-H-2Kb and anti-H-2Kb monoclonal antibodies, and analyzed in flow cytometry. (TIFF) [file pone.0044244.s004.tiff]

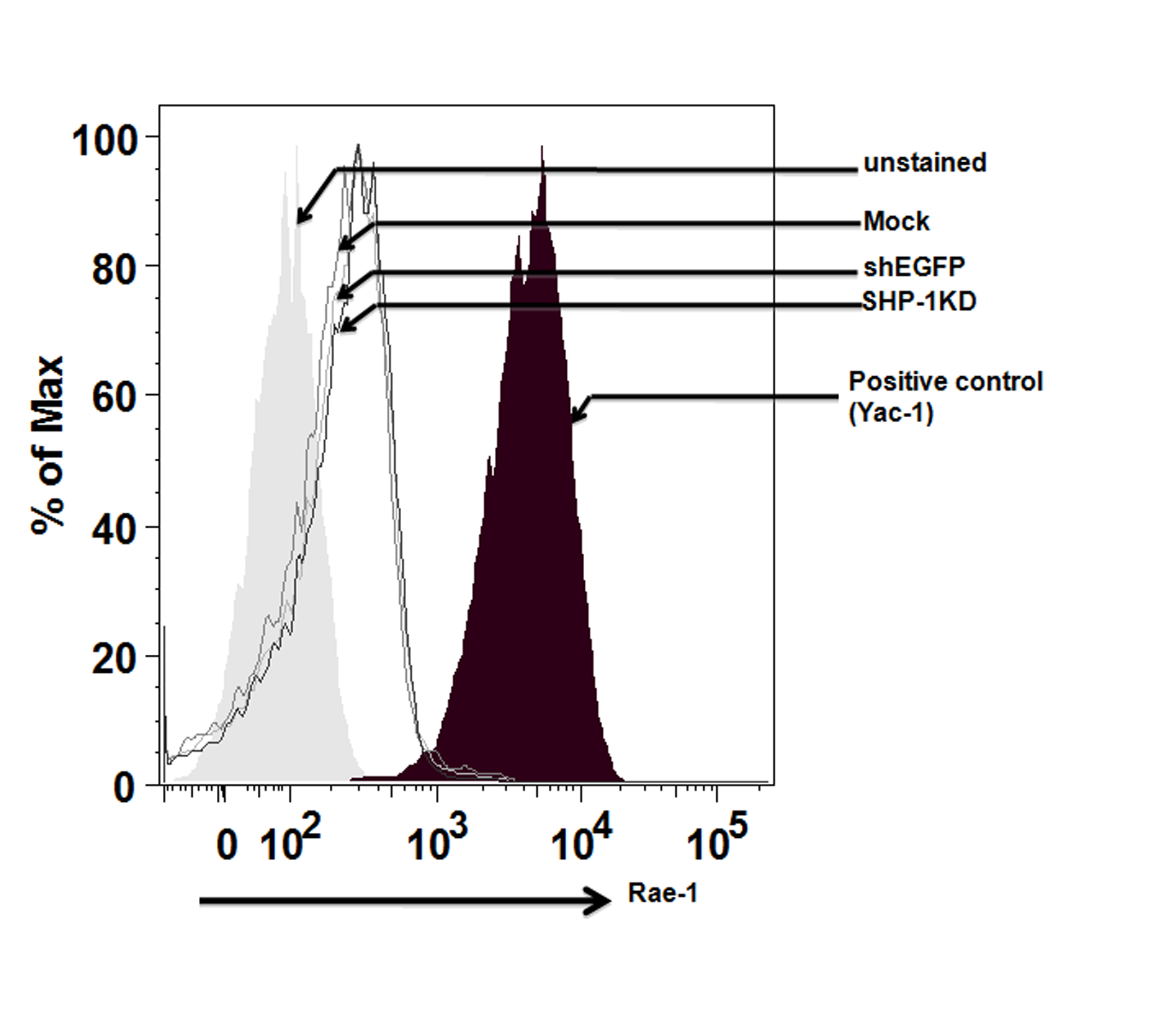

Supplement: Figure S5 — SHP-1 knockdown NK cells exhibited comparable Rae-1 expression. Mock, shEGFP-transduced and SHP-1 shRNA-transduced NK cells were surface stained for the expression of Rae-1 in flow cytometry. SHP-1 knockdown NK-cells, when compared to the mock and shEGFP- transduced controls, demonstrated no observable difference in their Rae-1 expression. YAC-1 cells were used as positive control of Rae-1 staining. (TIFF) [file pone.0044244.s005.tiff]
